# Switching efavirenz to rilpivirine in virologically suppressed adolescents with HIV: a multi‐centre 48‐week efficacy and safety study in Thailand

**DOI:** 10.1002/jia2.25862

**Published:** 2022-01-09

**Authors:** Wanatpreeya Phongsamart, Watsamon Jantarabenjakul, Sasitorn Chantaratin, Suvaporn Anugulruengkitt, Piyarat Suntarattiwong, Pakpen Sirikutt, Pope Kosalaraksa, Alan Maleesatharn, Kulkanya Chokephaibulkit

**Affiliations:** ^1^ Department of Pediatrics Faculty of Medicine Siriraj Hospital Mahidol University Bangkok Thailand; ^2^ Department of Pediatrics Faculty of Medicine Chulalongkorn University Bangkok Thailand; ^3^ Thai Red Cross AIDS Research Center (TRCARC) HIVNAT Bangkok Thailand; ^4^ Division of Child and Adolescent Psychiatry Faculty of Medicine Siriraj Hospital Mahidol University Bangkok Thailand; ^5^ Queen Sirikit National Institute of Child Health Bangkok Thailand; ^6^ Department of Pediatrics Faculty of Medicine Khon Kaen University Khon Kaen Thailand

**Keywords:** adolescents, efavirenz, HIV, rilpivirine, treatment switch

## Abstract

**Introduction:**

Efavirenz (EFV) is commonly used for first‐line antiretroviral therapy in children and adolescents with HIV, but is associated with neuropsychiatric and metabolic side effects. Rilpivirine (RPV) is better tolerated, and switching from EFV to RPV in virologically suppressed adults has been safe and efficacious, but data in adolescents are limited. Our primary objective was to describe the 48‐week immunologic and virologic outcomes in virologically suppressed adolescents switching from EFV‐ to RPV‐based antiretroviral therapy. Secondary objectives included assessment of neuropsychiatric adverse events, quality of life (QOL) and metabolic profiles while on RPV.

**Methods:**

We conducted an open‐label, single‐arm, multi‐centre study in Thailand in virologically suppressed adolescents aged 12–18 years receiving EFV plus two nucleoside/tide reverse transcriptase inhibitors (NRTIs/NtRTI) for ≥3 months. Participants were switched to an RPV (25 mg) tablet once daily, with the same NRTIs. HIV RNA viral load, CD4 cell count, fasting total cholesterol (TC), triglyceride, glucose, neuropsychiatric adverse events, depression and QOL were assessed over 48 weeks. Data were collected between February 2016 and September 2018.

**Results:**

One hundred and two (52% male) adolescents were enrolled. Median age at entry was 15.5 years (IQR 14.4–17.0), median CD4 count was 664 cells/mm^3^ (29.9%); 58% were receiving tenofovir‐DF and emtricitabine. At weeks 24 and 48, 96 (94.1%) and 94 (92.2%) participants were virologically suppressed, respectively, with no significant change in CD4 cell counts from baseline. Six (5.9%) participants experienced virologic failure, two of whom had RPV‐associated mutations (K101E and Y181C) and a lamivudine‐associated mutation (M184V/I). There were significant decreases in TC, triglyceride, high‐density lipoprotein (HDL) and low‐density lipoprotein (LDL) at weeks 24 and 48 and a significant increase in LDL/HDL ratio at week 48 compared to baseline. No substantial changes in EFV‐related symptoms, depression score or health‐related QOL were observed over time; however, there was significant improvement in performance‐based assessments of executive function at week 24.

**Conclusions:**

A high proportion of adolescents (>92%) remained virologically suppressed up to 48 weeks after switching from EFV to RPV along with no significant change in CD4 cell counts. RPV was well tolerated and associated with improvements in metabolic profiles and executive function.

## 1 INTRODUCTION

Antiretroviral therapy (ART) has substantially reduced mortality and improved the long‐term prognosis for infants and children living with HIV. Today, the majority of children living with HIV are surviving into adolescence [1] and optimizing therapy, primarily through regimen simplification and limiting drug‐related side effects, which are valuable tools to facilitate drug adherence during this often‐challenging period of child development.

Efavirenz (EFV)‐based ART has been the cornerstone of first‐line treatments in adults, adolescents and children older than 3 years in resource‐limited settings since 2016 [2]. EFV has shown good virological efficacy, but has been associated with neuropsychiatric and metabolic side effects that can lead to the discontinuation of therapy [3]. Common central nervous system (CNS) side effects associated with EFV treatment include vivid dreams, dizziness, headache and depression, but in most individuals, these symptoms resolve over the first few weeks of treatment [4]. However, long‐term neuropsychiatric effects can occur, with higher rates of suicidality (i.e. reported suicidal ideation and attempted/completed suicide) among individuals treated with EFV‐based ART [5]. Elevations in total cholesterol (TC) and triglycerides have also been associated with EFV use [6]. Thus, alternative options to EFV are needed for those experiencing or at high risk of EFV‐related side effects.

Rilpivirine (RPV) is a second‐generation non‐nucleoside reverse transcriptase inhibitor (NNRTI) available in Thailand for free through the national ART program. The efficacy and safety of RPV is well established in adults. Among ART‐naive adults with a baseline RNA viral load (VL) <100,000 copies/ml, the virologic efficacy of RPV was non‐inferior to EFV over 48 weeks, and RPV had a more favourable tolerability profile, with lower rates of CNS toxicity and lipid abnormalities compared to EFV [7, 8, 9]. Switching from EFV‐ to RPV‐based ART in virologically suppressed adults was also found to be safe and efficacious up to 48 weeks, with 93.9% remaining suppressed and no subjects stopping treatment due to adverse events [10]. A study in adolescents using adult doses revealed RPV plasma exposures comparable to adults along with good short‐term safety and antiviral activity [11]. The PAINT study assessed the safety and efficacy of RPV‐based ART in treatment‐naïve adolescents, and among those with a baseline VL ≤100,000 copies/ml, 79% achieved VL <50 copies/ml at week 48. RPV resistance‐associated mutations (RAMs) were detected in five of eight subjects who experienced virologic failure, and adverse events considered possibly related to treatment were mostly somnolence and nausea but generally mild in this adolescent population [12].

Overall, RPV is a potential alternative to standard EFV‐based ART for adolescents struggling with mild CNS side effects, or to help reduce the risk of metabolic side effects associated with long‐term EFV use. Our primary objective was to describe the 48‐week immunologic and virologic outcomes in virologically suppressed adolescents switching from EFV‐ to RPV‐based ART. Assessment of neuropsychiatric adverse events, quality of life (QOL) and metabolic profiles while on RPV were secondary objectives.

## 2 METHODS

We performed an open‐label, single‐arm, multi‐centre study in adolescents with HIV aged 12–18 years old. This study was performed at four sites in Thailand: Siriraj Hospital, Queen Sirikit National Institute of Child Health, HIV‐NAT Chulalongkorn University and Khon Kaen University. Data were collected between 10th February 2016 and 19th September 2018.

Prior to screening, caregivers provided written informed consent and adolescents who knew their HIV status provided written assent. Adolescents 12–18 years, with a body weight ≥25 kilograms and receiving ART composed of EFV plus two nucleoside or nucleotide reverse transcriptase inhibitors [N(t)RTI]) for ≥3 months with virological suppression (HIV RNA <50 copies/ml within the last 12 months) were screened for eligibility. We excluded individuals with prior evidence of NNRTI‐associated resistance mutations based on the IAS–USA HIV drug‐resistance mutations list (2019) (V90I, A98G, L100I, K101E/H/P/Q/R/N, K103N/S, V106A/M/I, V108I, E138K/A/G/Q/R, V179D/F/L/T, Y181C/I/V, Y188L/C/H, G190A/S/E,H221Y, P225H, F227L/C/R, M230L/I and L234I) [13]. However, resistance genotyping was not always obtained and adolescents who may have failed on a prior NNRTI regimen were consequently also excluded if no historical genotype result was available. In addition, adolescents were excluded if they were currently receiving an HIV protease inhibitor, were pregnant, had an alanine aminotransferase (ALT) ≥200 IU/L over the last 12 months, active opportunistic infection(s) related to immunosuppression, or significant medical problems in the investigator's opinion that would compromise participation, or concomitant treatment with drugs known to effect the pharmacokinetics of RPV (i.e. carbamazepine, phenobarbital, phenytoin, rifampicin, rifabutin, omeprazole, esomeprazole, lansoprazole, erythromycin, clarithromycin, azithromycin and roxithromycin).

At the study entry visit, EFV‐based ART was discontinued and switched to RPV‐based ART. Study participants were advised to take a single RPV 25 mg tablet once daily with a substantial meal to increase drug absorption. This food requirement was emphasized at each study visit. The choice of the NRTIs/NtRTI backbone was according to the standard of care in Thailand and inclusive of drugs and formulations provided for free through the national ART program. Study participants were also advised to report any adverse events (e.g. rash, insomnia, headache, etc.) that developed after switching to RPV. Due to the extended inductive effect of EFV on CYP3A4, there were initial questions around the potential for reduced RPV exposures immediately after switching from EFV; thus, at the start of this study, a pharmacokinetic sub‐study was performed in 20 participants. We have previously reported that RPV exposures were adequate in this sub‐cohort immediately after switching from EFV and study‐related dosing was not altered [14]. The planned duration of follow‐up for each study participant was 48 weeks. HIV‐1 RNA VL was performed at entry, weeks 12, 24 and 48. Complete blood count, CD4 cell counts and percentage, ALT and creatinine were assessed at baseline, weeks 4, 24 and 48. Fasting TC, low‐density lipoprotein (LDL), high‐density lipoprotein (HDL), triglyceride and glucose were tested at baseline, weeks 24 and 48. These laboratory tests were collected to monitor the safety of RPV. If a participant's VL was found to be >50 copies/ml, the VL test was repeated within 4–8 weeks and adherence counselling provided. Virologic failure was defined as a confirmed VL >50 copies/ml during the 48‐week follow‐up. If a VL >1000 copies/ml was reported, an HIV genotypic‐resistance test (GRT) was also performed. Resistance results were interpreted using the 2019 edition of the IAS–USA HIV drug‐resistance mutations list [13]. Modification of ART due to either drug resistance and/or safety considerations was at the discretion of the site investigators. Executive function and global cognition were evaluated by standard tests, including the non‐verbal part of the Standard Progressive Matrices (SPM), digit symbol coding sub‐test of the Wechsler Intelligence Scale for Children‐Third Edition (WISC‐III), Trail Making Test (TMT) part A and part B, and the Wisconsin Card Sorting Test (WCST) at baseline and week 24. Depression, QOL and EFV‐related symptoms were assessed at baseline, weeks 4 and 24 by the Center for Epidemiologic Studies‐Depression Scale (CES‐D), PedsQL^TM^ 4.0 [15, 16] and a self‐reported questionnaire, respectively.

This study was approved by the Institutional Review Board (IRB) of the Faculty of Medicine Siriraj Hospital, Mahidol University, Queen Sirikit National Institute of Child Health Chulalongkorn University and Khon Kaen University.

The primary objective was to describe the 48‐week virologic and immunologic outcomes in virologically suppressed adolescents switching from EFV‐ to RPV‐based ART. Under the assumption that all study participants were virologically suppressed at the time of treatment switch, and that 10% would experience virological failure over 48 weeks [17], a sample size of 100 subjects would provide 90% power to detect a change of 10% from baseline based on McNemar's test of paired proportions for a single group of subjects (two‐sided, alpha of 0.05). The primary endpoints were the proportion of study participants with VL <50 copies/ml and change of CD4 cell count from baseline at 48 weeks. Analysis for virologic suppression was intention‐to‐treat, with study losses to follow‐up or deaths imputed as failures. Both primary endpoints were analysed using the Wilcoxon signed‐rank test. The secondary endpoints of change in TC, LDL, HDL, triglyceride, LDL/HDL ratio, TC/HDL ratio and glucose were compared between baseline and week 24, and baseline and week 48. In addition, depression, QOL and EFV‐related symptom scores were compared between baseline and week 4, and baseline and week 24. Each of these comparisons were tested using a Wilcoxon signed‐rank test. Changes in executive function and global cognition were compared between baseline and week 24 by a McNemar test. Participant data were collected from source documents onto Case Record Forms, and transferred to an electronic data capture program. All analyses were performed using STATA, version 11.2 (StataCorp, College Station, TX, USA).

## 3 RESULTS

**Figure 1:**
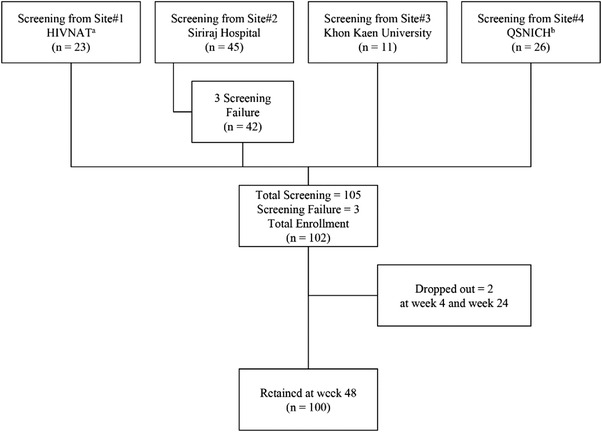
CONSORT diagram of switching efavirenz to rilpivirine in virologically suppressed adolescents with HIV: a multi‐centre 48‐week efficacy and safety study in Thailand. Abbreviations: HIV‐NAT, The HIV Netherlands Australia Thailand Research Collaboration; QSNICH, Queen Sirikit National Institute of Child Health.

A total of 105 adolescents were screened and 102 (52% male) were enrolled (Figure 1). The three adolescents who did not enrol had a VL >50 copies/ml at screening. All study participants were receiving first‐line EFV‐based ART at enrolment. Baseline demographic data of study participants are shown in Table 1. The median age at enrolment was 15.5 years [interquartile range (IQR) 14.4–17.0] with a median CD4 count of 664 cells/mm^3^ (IQR 553–862) (29.9%). Twenty‐seven (26.5%) participants were receiving a single‐tablet regimen of tenofovir‐DF/emtricitabine/EFV (TDF/FTC/EFV) before switching ART. The majority of study participants (58%) were receiving a tenofovir‐DF/emtricitabine (TDF/FTC)‐backbone.

**Table 1:** Baseline demographic data of study participants in switching efavirenz to rilpivirine in virologically suppressed adolescents with HIV: a 48‐week efficacy and safety, multi‐centre study in Thailand (*n* = 102)

Characteristics	*N* = 102
Enrolment by site; *n* (%)	
The HIV Netherlands Australia Thailand Research Collaboration	23 (22.5)
Khon Kaen University	11 (10.8)
Queen Sirikit National Institute of Child Health	26 (25.5)
Siriraj Hospital	42 (41.2)
Sex; *n* (%)	
Female	49 (48.0)
Male	53 (52.0)
Age (years); median (IQR)	15.6 (14.4–17.0)
WHO Stage; *n* (%)	
Stage 1	22 (21.6)
Stage 2	18 (17.7)
Stage 3	46 (45.1)
Stage 4	16 (15.6)
CDC Stage; *n* (%)	
N	12 (11.8)
A	21 (20.6)
B	33 (32.4)
C	36 (35.3)
Nadir CD4 percentage; median (IQR)	12.3 (3.0–20.0)
Nadir CD4 cells/mm^3^; median (IQR)	289 (46–493)

Abbreviations: CDC, Centers for Disease Control and Prevention; IQR, interquartile range; WHO, World Health Organization.

RPV was well tolerated during the follow‐up period. No study participant experienced any treatment‐emergent adverse event that led to temporary interruption or permanent discontinuation of the study drug. Two (2.0%) study participants dropped out, one due to pregnancy (week 24) and another following the advice of their local IRB due to being underweight (week 4). Over the 48 weeks of follow‐up, five participants had detectable VL in the range of 51–202 copies/ml. Of these, four became virologically suppressed after enhanced adherence counselling, while one who had a VL of 202 copies/ml at week 48 was subsequently lost to follow‐up. Overall, 96 (94.1%) and 94 (92.2%) of 102 participants maintained virologic suppression at weeks 24 and 48, respectively. No significant change in CD4 cell counts at weeks 24 and 48 from baseline was observed (Table 2). Among the six study participants who met the virologic failure criteria (confirmed VL >50 copies/ml), two (33.3%) had RPV‐associated mutations: K101E (*n* = 2) and Y181C (*n* = 1). Drug‐resistance mutations detected among study participants with RPV‐associated mutations included: participant #1 NNRTI mutations –– K101E, K103N, Y181C, G190A, N348I and NRTI mutation M184V; participant #2 NNRTI mutation K101E and NRTI mutation M184I. Two study participants experiencing virologic failure also had NRTI mutations. Two other study participants developed the V179D mutation, which is not associated with reduced susceptibility to RPV on its own. Significant decreases in TC, triglyceride, HDL and LDL were observed at weeks 24 and 48 compared to baseline (*p* <0.001, Table 2). The TC/HDL ratio at weeks 24 and 48 increased compared to baseline, and the LDL/HDL ratio increased at week 24 compared to baseline, although neither change reached statistical significance. However, there was a significant increase in the LDL/HDL ratio at week 48 compared to baseline (*p* = 0.016, Table 2). EFV‐related symptoms were evaluated by self‐report, and no significant changes in sleep, dizziness/headache, concentration, mood/emotion and total scores were observed. There was no significant change in health‐related QOL, including physical functioning, emotional wellbeing, social and school functioning evaluated by PedsQL either by self‐report or parent‐proxy report for participants. There was no significant change in the depression score by CES‐D (Table 3).

**Table 2:** Comparison of virologic, immunologic and metabolic outcomes between baseline versus week 24, and baseline versus week 48 after switching from efavirenz to rilpivirine in virologically suppressed adolescents with HIV in Thailand (*n* = 102)

Laboratory results	Week 0	Week 12	Week 24	Week 48	*p*‐Value Week 0 versus week 24	*p*‐Value Week 0 versus week 48
Virology						
Virological suppression (HIV RNA <50 copies/ml); *n* (%)	102 (100.0)	94 (92.2)	96 (94.1)	94 (92.2)	0.031	0.008
Immunology						
CD4 cells/mm^3^; median (IQR)	664 (553–862)	–	689 (565–859)	667 (553–920)	0.219	0.866
Lipid profile; median (IQR)						
Cholesterol	159 (144–176)	–	140 (123–156)	139 (126–159)	<0.001	<0.001
Triglycerides	83 (68–110)	–	70 (59–87)	74 (60–85)	<0.001	<0.001
HDL	52 (41–62)	–	43 (37–54)	44 (38–52)	<0.001	<0.001
LDL	89 (74–104)	–	79 (64–92)	81 (68–95)	<0.001	<0.001
LDL/HDL ratio	1.8 (1.3–2.2)	–	1.8 (1.4–2.4)	1.9 (1.5–2.3)	0.409	0.016
Cholesterol/HDL ratio	3.2 (2.7–3.7)	–	3.2 (2.6–3.7)	3.3 (2.8–3.7)	0.933	0.249
Glucose	86 (81–91)	–	85 (81–91)	85 (80–91)	0.721	0.166

Abbreviations: HDL, high‐density lipoprotein; IQR, interquartile range; LDL, low‐density lipoprotein.

**Table 3:** Comparison of health‐related quality of life evaluated by self‐report, PedsQL, and depression evaluated by CES‐D at baseline and week 24 after switching from efavirenz to rilpivirine in virologically suppressed adolescents with HIV in Thailand (*n* = 102)

Tests	Week 0	Week 4	Week 24	*p*‐Value Week 0 versus week 4	*p*‐Value Week 0 versus week 24
Self‐report, median (IQR)					
Sleep	3 (1–6)	4 (1–7)	4 (1–7)	0.152	0.237
Dizziness/headache	1 (0–3)	2 (0–3)	2 (0–3)	0.797	0.638
Concentration	2 (1–2)	2 (1–3)	2 (1–3)	0.146	0.225
Mood/emotion	5 (4–7)	5 (4–7)	5 (4–8)	0.617	0.522
PedsQL					
Parents report, median (IQR)					
Health problem	4 (0–15)	4 (0–14)	6 (0–17)	0.688	0.577
Emotional problem	3 (0–6)	3 (0–8)	4 (0–7)	0.843	0.952
Social problem	1 (0–4)	1 (0–5)	2 (0–4)	0.358	0.717
School problem	6 (2–9)	4 (2–9)	7 (2–9)	0.231	0.816
Child report, median (IQR)					
Health problem	4 (1–9)	3 (0–8)	3 (0–7)	0.152	0.246
Emotional problem	4 (1–7)	3 (1–8)	4 (1–7)	0.216	0.999
Social problem	0 (0–4)	1 (0–3)	0 (0–2)	0.510	0.279
School problem	6 (3–8)	6 (3–9)	5 (3–8)	0.717	0.253
CES‐D					
Total score	15 (11–18)	15 (11–19)	14 (11–19)	0.779	0.767
Normal (≤22); *n* (%)	87 (85.3)	84 (83.2)	85 (85.0)		
Abnormal (>22); *n* (%)	15 (14.7)	17 (16.8)	15 (15.0)		

Abbreviations:

CES‐D, Center for Epidemiologic Studies‐Depression Scale;

IQR, interquartile range; PedsQL^TM^, Pediatric Quality of Life Inventory^TM^ 4.0.

Of 100 participants who were able to complete the neuropsychological assessment, 19 (19.0%) participants and 1 (1.0%) participant were slow learner and had intellectual disability evaluated by SPM, respectively. In addition, 41.0%, 54.0% and 68.7% of participants scored lower than average as measured by coding, TMT Part A and Part B, respectively (Supporting Information). At week 24, there was a significant improvement in performance‐based assessments of executive and cognitive function by coding, TMT Part A, and Part B after switching to RPV from EFV (Table 4). The proportion of subjects classified with normal IQ remained the same 24 weeks after switching RPV from EFV.

**Table 4:** Comparison of executive and cognitive function at baseline and week 24 after switching from efavirenz to rilpivirine in virologically suppressed adolescents with HIV in Thailand

Week 0 *n* (%)^a^		Week 24 *n* (%)^a^		*p*‐Value
Standard Progressive Matrices	Total^b^	Normal	Abnormal	0.549
Normal	78 (100.0)	74 (94.9)	4 (5.1)	
Abnormal	19 (100.0)	7 (36.8)	12 (63.2)	
Total	97	81	16	
Coding	Total	Average to superior	Below average	0.049
Average to superior	57 (100.0)	53 (93.0)	4 (7.0)	
Below average	40 (100.0)	13 (32.5)	27 (67.5)	
Total	97	66	31	
Trail Making Test: part A	Total	Average to fast	Slow	<0.001
Average to fast	45 (100.0)	41 (91.1)	4 (8.9)	
Slow	52 (100.0)	30 (57.7)	22 (42.3)	
Total	97	71	26	
Trail Making Test: part B	Total	Average to fast	Slow	0.001
Average to fast	31 (100.0)	25 (80.6)	6 (19.4)	
Slow	65 (100.0)	24 (36.9)	41 (63.1)	
Total	96	49	47	
WCST: total number of errors	Total	Average to fast	Slow	0.103
Average to above	48 (100.0)	40 (83.3)	8 (16.7)	
Below average to impairment	45 (100.0)	16 (35.6)	29 (64.4)	
Total	93	56	37	

Abbreviation: WCST, Wisconsin Card Sorting Test.

^a^ Percentage by row.

^b^ Only those with available test results at baseline and week 24 were included in the analysis.

## 4 DISCUSSION

We found that a high proportion of virologically suppressed adolescents switching from EFV‐ to RPV‐based ART maintained virologic suppression after 48 weeks. This finding is important as there is a paucity of data about RPV in adolescents. Over 92% of the adolescents maintained virologic suppression up to 48 weeks. Our results are comparable to a 48‐week, phase 2b study in adults switching from EFV/TDF/FTC to RPV/TDF/FTC, which reported 93.9% of participants remaining virologically suppressed [10]. While no emergence of RPV resistance was observed in the 4.1% of study participants with virologic failure in the adult study, two of six adolescents with virologic failure in our study developed RPV RAMs (K101E and Y181C) and a lamivudine‐associated mutation (M184V/I). In ART‐naïve adults initiating RPV/FTC/TDF, resistance mutations to RPV development were more frequent in subjects with baseline HIV‐1 RNA >100,000 copies/ml compared to baseline HIV‐1 RNA ≤ 100,000 copies/ml [18]. Resistance analyses of RPV from the ECHO and THRIVE Phase III trials found that 6.7% of subjects on RPV developed treatment‐emergent NNRTI RAMs, with E138K+M184I being the most frequent combination [19].

Consistent with studies in adults, an important aspect of the current study is that switching from EFV‐ to RPV‐based ART was extremely well tolerated with no treatment discontinuations due to adverse events. Among virologically suppressed adults switching from EFV/FTC/TDF to RPV/FTC/TDF, there were significant decreases in fasting TC, direct LDL cholesterol and triglycerides at week 12, and these changes persisted through week 48 [10]. Switching from EFV‐based to RPV‐based ART among virologically suppressed adolescents in our study was associated with improvements in metabolic profiles with significant decreases in TC, triglyceride and LDL at weeks 24 and 48. There was a significant decrease in HDL at weeks 24 and 48. However, the median (IQR) HDL at weeks 24 and 48 was 43 (37–54) and 44 (38–52) mg/dl, respectively, which was in the range of borderline level of HDL (35–45 mg/dl), as defined by the National Cholesterol Education Program [20]. While there was a statistically significant increase in the LDL/HDL ratio at week 48, the magnitude of the increase was small and not considered clinically significant. The change in TC/HDL ratio was not statistically significant, and the median ratio was below the cut‐off associated with development of metabolic syndrome reported in Korean adolescents; in which a high risk of metabolic syndrome was associated with a TC/HDL ratio ≥3.8 [OR: 14.8 (95% CI 2.8–77.4)] and those with a TG/HDL ratio ≥3.3 [OR: 30.6 (95% CI, 6.0–157.6)] [21]. When TC/HDL and TG/HDL were both above the cut‐off values, the risk of metabolic syndrome further increased [OR: 36.2 (95%, 7.2–186.2)]. The significance and long‐term effects of these findings need further study.

Perhaps unexpectedly, we did not demonstrate a significant change in EFV‐related side effects evaluated by self‐report (i.e. sleep, dizziness/headache, concentration, mood/emotion and total scores). An observational study in Tanzania compared the competence (social involvement, activities and school performance), psychopathology (internalizing and externalizing problems) and cognitive performance (intelligence and working memory) of children with HIV aged 6–12 years receiving an EFV‐based versus non‐EFV‐based regimens, and found that EFV use in children was associated with a mild increase in neuropsychiatric symptoms [22]. The lack of a difference in our study could be explained by selection bias, as the study participants enrolled were stable on EFV‐based ART (for ≥3 months) and therefore somewhat tolerant of EFV, while those who did not tolerate EFV may have already been switched to an alternative regimen. Furthermore, it is possible that children and adolescents may tolerate EFV better than adults and report fewer neuropsychiatric side effects. Improvements in neuropsychiatric symptoms, sleep quality and self‐perceived cognition were observed in a randomized study in adults who already had altered neurocognitive assessment, depression, anxiety or low sleep‐quality, switching either immediately or delayed to RPV‐ from EFV‐based ART [23]. Interestingly, switching to RPV was not found to improve cognitive function in these adults. In our study, 20.0–68.7% of adolescents had some degree of impairment of executive and cognitive function at baseline, and significant improvement in executive function was observed. A study on executive function and emotional behavioural problems in a cohort of Asian adolescents living with HIV, in which 86.4% had virologic suppression, found that there were significantly higher rates of impairment in all assessed measures of executive function and behavioural problems in adolescents living with HIV, compared with HIV‐unexposed, uninfected youth, after adjustment for relevant socio‐demographic factors [24]. Thai adolescents living with HIV on ART (with 93% achieving virologic suppression) had significantly lower scores on the measures of executive function compared to HIV‐uninfected controls across multiple neuropsychological tests [25]. These findings are consistent with prior studies in the U.S.‐based Pediatric HIV/AIDS Cohort [26]. Individual differences in executive function are associated with multiple important aspects of human health, including academic and occupational functioning, interpersonal problems, substance use, physical health and mental health. Of critical importance, executive function impairments are associated with poor academic performance as well as emotional and behavioural problems [27]. A key finding of our study is the significant improvement in performance‐based assessments of executive and cognitive function at week 24 after switching to RPV from EFV.

We demonstrated that RPV was well tolerated and was associated with improvement in metabolic profiles, executive and cognitive function, and is a reasonable alternative for adolescents experiencing EFV‐associated side effects. Of note, adolescents must be able to take RPV following a regular schedule with a full meal to assure adequate RPV concentrations, which may limit its usefulness for adolescents with irregular eating schedules. Importantly, our findings support the use of RPV as a part of a long‐acting injectable treatment regimen, which would be an ideal option for adolescents [28].

Our study had several limitations. Firstly, an HIV GRT was not performed prior to initiation of ART. This is consistent with the national antiretroviral treatment guidelines in Thailand. Although GRT could have identified those less likely to respond to RPV, we only enrolled participants who had not previously failed NNRTI‐based ART (based on virologic criteria). Therefore, the presence of NNRTI mutations was expected to be very low and not anticipated to affect treatment outcomes. Secondly, we did not record participant adherence to ART – whether by self‐report or other means (e.g. pill count), which could have been a primary contributor to virologic failure. Thirdly, improvement on repetition of neurocognitive testing could have been due to learning the skills of the test over time. Furthermore, there were many potential psychosocial issues among adolescents living with HIV, which may have influenced the results of the QOL and depression scores while on RPV.

Recently, the World Health Organization revised its recommendations for preferred first‐ and second‐line regimens for adolescents and adults to include dolutegravir (DTG)‐based ART [29]. The Thailand National Guidelines on HIV/AIDS Diagnosis, Treatment and Prevention 2020/2021 also revised its recommendations accordingly. RPV is now recommended as an alternative first‐line regimen for those with VL <500,000 copies/ml, or CD4 >350 cells/mm^3^ if the HIV RNA VL is not available. Furthermore, RPV can be used for a treatment switch for those individuals who have had an undetectable VL for 6–12 months and do not have prior NNRTI resistance, or those who cannot tolerate EFV within 2 weeks after initiation [30]. Although our data were collected between 2016 and 2018, they remain relevant more broadly in Asia today. To date, access to DTG remains limited in many countries in the region, and rollout has been slower overall than in African countries – a challenge that has been exacerbated by the impact of the COVID‐19 pandemic on drug production and supply chains [31]. Until DTG is widely available, alternatives to EFV, such as RPV, are still needed.

## 5 CONCLUSIONS

Overall, the majority of adolescents on EFV‐based ART with HIV‐VL <50 copies/ml who switched to RPV remained virologically suppressed up to 48 weeks, along with no significant change in CD4 cell counts. Among the few participants with virologic failure, development of RPV‐RAMs (K101E and Y181C) was infrequent. RPV was well tolerated and was associated with improvement in metabolic profiles, executive and cognitive function and is a reasonable alternative for adolescents experiencing EFV‐associated side effects.

## COMPETING INTERESTS

The authors have no competing interests.

## AUTHORS’ CONTRIBUTIONS

Conceptualization: WP, KC and SC. Investigation: WP, WJ, SC, SA, PS, PS, PS and KC. Data analysis: AM. Writing – original draft: WP, SC and AM. Writing – review and editing: KC, WJ, SA, PS, PS and PK. Funding acquisition: WP and KC.

## FUNDING

This study was supported by grants from amfAR, the Foundation for AIDS Research (TREAT Asia program) and the Faculty of Medicine Siriraj Hospital, Mahidol University (grant number R015836002).

## Data Availability

The data are available upon request to the corresponding author.

## References

[1] Mofenson LM , Cotton MF . The challenges of success: adolescents with perinatal HIV infection. J Int AIDS Soc. 2013; 16:18650. 23782484 10.7448/IAS.16.1.18650 PMC3687076

[2] World Health Organization . Consolidated guidelines on the use of antiretroviral drugs for treating and preventing HIV infection. Recommendations for a public health approach. 2nd ed. 2016. Available from https://www.who.int/hiv/pub/arv/arv‐2016/en/. Accessed 11 March 2021. 27466667

[3] Mbuagbaw L , Mursleen S , Irlam JH , Spaulding AB , Rutherford GW , Siegfried N . Efavirenz or nevirapine in three‐drug combination therapy with two nucleoside or nucleotide‐reverse transcriptase inhibitors for initial treatment of HIV infection in antiretroviral‐naïve individuals. Cochrane Database Syst Rev. 2016; 12:CD004246. 27943261 10.1002/14651858.CD004246.pub4 PMC5450880

[4] Kenedi CA , Goforth HW . A systematic review of the psychiatric side‐effects of efavirenz. AIDS Behav. 2011; 15:1803–18. 21484283 10.1007/s10461-011-9939-5

[5] Mollan KR , Smurzynski M , Eron JJ , Daar ES , Campbell TB , Sax PE , et al. Association between efavirenz as initial therapy for HIV‐1 infection and increased risk for suicidal ideation or attempted or completed suicide: an analysis of trial data. Ann Intern Med. 2014; 161:1–10. 24979445 10.7326/M14-0293 PMC4204642

[6] Ward DJ , Curtin JM . Switch from efavirenz to nevirapine associated with resolution of efavirenz‐related neuropsychiatric adverse events and improvement in lipid profiles. AIDS Patient Care STDs. 2006; 20:542–8. 16893323 10.1089/apc.2006.20.542

[7] Cohen CJ , Molina JM , Cahn P , Clotet B , Fourie J , Grinsztejn B , et al. Efficacy and safety of rilpivirine (TMC278) versus efavirenz at 48 weeks in treatment‐naive HIV‐1‐infected patients: pooled results from the phase 3 double‐blind randomized ECHO and THRIVE Trials. J Acquir Immune Defic Syndr. 2012; 60:33–42. 22343174 10.1097/QAI.0b013e31824d006e

[8] Molina JM , Clumeck N , Redant K , Rimsky L , Vanveggel S , Stevens M , et al. Rilpivirine vs. efavirenz in HIV‐1 patients with baseline viral load 100,000 copies/ml or less: week 48 phase III analysis. AIDS. 2013; 27:889–97. 23276806 10.1097/QAD.0b013e32835e1554

[9] Cohen CJ , Molina JM , Cassetti I , Chetchotisakd P , Lazzarin A , Orkin C , et al. Week 96 efficacy and safety of rilpivirine in treatment‐naive, HIV‐1 patients in two phase III randomized trials. AIDS. 2013;27:939–50. 23211772 10.1097/QAD.0b013e32835cee6e

[10] Mills AM , Cohen C , Dejesus E , Brinson C , Williams S , Yale KL , et al. Efficacy and safety 48 weeks after switching from efavirenz to rilpivirine using emtricitabine/tenofovir disoproxil fumarate‐based single‐tablet regimens. HIV Clin Trials. 2013; 14:216–23. 24144898 10.1310/hct1405-216

[11] Crauwels H , Hoogstoel A , Vanveggel S , Yarnall W , Stevens M , Boven K , et al. Rilpivirine pharmacokinetics in HIV‐1‐infected adolescents: a substudy of PAINT (Phase II trial). In: 21st Conference on Retroviruses and Opportunistic Infections, March 3–6, 2014. Boston, MA.

[12] Lombaard J , Bunupuradah T , Flynn PM , Ramapuram J , Ssali F , Crauwels H , et al. Rilpivirine as a treatment for HIV‐infected antiretroviral‐naive adolescents: week 48 safety, efficacy, virology and pharmacokinetics. Pediatr Infect Dis J. 2016; 35:1215–21. 27294305 10.1097/INF.0000000000001275

[13] Wensing AM , Calvez V , Ceccherini‐Silberstein F , Charpentier C , Günthard HF , Paredes R , et al. 2019 Update of the drug resistance mutations in HIV‐1. Top Antivir Med. 2019; 27:111–21. 31634862 PMC6892618

[14] Jantarabenjakul W , Anugulruengkitt S , Kasipong N , Thammajaruk N , Sophonphan J , Bunupuradah T , et al. Pharmacokinetics of rilpivirine and 24‐week outcomes after switching from efavirenz in virologically suppressed HIV‐1‐infected adolescents. Antivir Ther. 2018; 23:259–65. 28994660 10.3851/IMP3198

[15] Punpanich W , Boon‐Yasidhi V , Chokephaibulkit K , Prasitsuebsai W , Chantbuddhiwet U , Leowsrisook P , et al. Health‐related Quality of Life of Thai children with HIV infection: a comparison of the Thai Quality of Life in Children (ThQLC) with the Pediatric Quality of Life Inventory version 4.0 (PedsQL 4.0) Generic Core Scales. Qual Life Res. 2010; 19:1509–16. 20730627 10.1007/s11136-010-9708-3 PMC2977060

[16] Clifford DB , Evans S , Yang Y , Acosta EP , Goodkin K , Tashima K , et al. Impact of efavirenz on neuropsychological performance and symptoms in HIV‐infected individuals. Ann Intern Med. 2005; 143:714–21. 16287792 10.7326/0003-4819-143-10-200511150-00008

[17] Fisher M , Palella FJ , Tebas P , Gazzard B , Ruane P , van Lunzen J , et al. SPIRIT: switching to emtricitabine/rilpivirine/tenofovir DF single‐tablet regimen from boosted protease inhibitor maintains HIV suppression at week 48. J Int AIDS Soc. 2012; 15:18275.

[18] Porter DP , Kulkarni R , Fralich T , Miller MD , White KL . 96‐Week resistance analyses of the STaR study: rilpivirine/emtricitabine/tenofovir DF versus efavirenz/emtricitabine/tenofovir DF in antiretroviral‐naive, HIV‐1‐infected subjects. HIV Clin Trials. 2015; 16:30–8. 25777187 10.1179/1528433614Z.0000000009

[19] Rimsky L , Van Eygen V , Hoogstoel A , Stevens M , Boven K , Picchio G , et al. 96‐Week resistance analyses of rilpivirine in treatment‐naive, HIV‐1‐infected adults from the ECHO and THRIVE Phase III trials. Antivir Ther. 2013; 18:967–77. 23714781 10.3851/IMP2636

[20] Expert Panel on Detection, Evaluation, and Treatment of High Blood Cholesterol in Adults . Executive Summary of the Third Report of the National Cholesterol Education Program (NCEP) Expert Panel on Detection, Evaluation, and Treatment of High Blood Cholesterol in Adults (Adult Treatment Panel III). JAMA. 2001; 285:2486–97. 11368702 10.1001/jama.285.19.2486

[21] Chu S , Jung J , Park M , Kim S . Risk assessment of metabolic syndrome in adolescents using the triglyceride/high‐density lipoprotein cholesterol ratio and the total cholesterol/high‐density lipoprotein cholesterol ratio. Ann Pediatr Endocrinol Metab. 2019; 24:41–8. 30943679 10.6065/apem.2019.24.1.41 PMC6449623

[22] Van de Wijer L , McHaile DN , de Mast Q , Mmbaga BT , Rommelse NNJ , Duinmaijer A , et al. Neuropsychiatric symptoms in Tanzanian HIV‐infected children receiving long‐term efavirenz treatment: a multicentre, cross‐sectional, observational study. Lancet HIV. 2019; 6:e250–8. 30770324 10.1016/S2352-3018(18)30329-1

[23] Lapadula G , Bernasconi DP , Bai F , Focà E , Di Biagio A , Bonora S , et al. Switching from efavirenz to rilpivirine improves sleep quality and self‐perceived cognition but has no impact on neurocognitive performances. AIDS. 2020;34:53–61. 31567160 10.1097/QAD.0000000000002377

[24] Kerr SJ , Puthanakit T , Malee KM , Thongpibul K , Ly PS , Sophonphan J , et al. Increased risk of executive function and emotional behavioral problems among virologically well‐controlled perinatally HIV‐infected adolescents in Thailand and Cambodia. J Acquir Immune Defic Syndr. 2019; 82:297–304. 31335589 10.1097/QAI.0000000000002132 PMC6814288

[25] Patel PB , Belden A , Handoko R , Puthanakit T , Kerr S , Kosalaraksa P , et al. Behavioral impairment and cognition in Thai adolescents affected by HIV. Glob Ment Health (Camb). 2021; 8:e3.26. 34026234 10.1017/gmh.2021.1 PMC8127634

[26] Nichols SL , Chernoff MC , Malee KM , Sirois PA , Woods SP , Williams PL , et al. Executive functioning in children and adolescents with perinatal HIV infection and perinatal HIV exposure. J Pediatr Infect Dis Soc. 2016; 5(suppl 1):S15–23. 10.1093/jpids/piw049 PMC5181544 27856672

[27] Snyder HR , Miyake A , Hankin BL . Advancing understanding of executive function impairments and psychopathology: bridging the gap between clinical and cognitive approaches. Front Psychol. 2015; 6:328. 25859234 10.3389/fpsyg.2015.00328 PMC4374537

[28] Rajoli RKR , Back DJ , Rannard S , Meyers CF , Flexner C , Owen A , et al. In silico dose prediction for long‐acting rilpivirine and cabotegravir administration to children and adolescents. Clin Pharmacokinet. 2018; 57:255–66. 28540638 10.1007/s40262-017-0557-x PMC5701864

[29] World Health Organization . Updated recommendations on first‐line and second‐line antiretroviral regimens and post‐exposure prophylaxis and recommendations on early infant diagnosis of HIV: interim guidelines. Supplement to the 2016 Consolidated Guidelines on the Use of Antiretroviral Drugs for Treating and Preventing HIV Infection. Geneva, Switzerland: World Health Organization; 2018.

[30] Department of Disease Control . Thai AIDS Society [Internet]. Thailand National Guidelines on HIV/AIDS Diagnosis, Treatment and Prevention. 2020. Available from: http://www.thaiaidssociety.org/images/PDF/thai_aids_guidelines_2020_2021.pdf. Accessed 5 August 2021.

[31] Drugs for Neglected Diseases initiative (DNDi) . Children and AIDS [Internet]. Accelerating access to optimal child‐friendly antiretroviral formulations for children living with HIV: lessons learned from eight sub‐Saharan African countries. 2020. Available from: https://www.childrenandaids.org/sites/default/files/2021‐05/ARV‐Lessons‐Learned‐Brief‐v5%20%281%29.pdf. Accessed 5 August 2021.

