# Supplementary material for: Switching efavirenz to rilpivirine in virologically suppressed adolescents with HIV: a multi‐centre 48‐week efficacy and safety study in Thailand
Source: J Int AIDS Soc. 2022 Jan 9;25(1):e25862. doi: 10.1002/jia2.25862 (PMC8743364; doi:10.1002/jia2.25862)
Supplement: Supplementary file 1 [file JIA2-25-e25862-s001.docx]

**Supplementary data**

Executive function and global cognitive function by standard neuropsychiatric tests evaluated at baseline and week 24 after switching efavirenz to rilpivirine in virologically-suppressed adolescents with HIV in Thailand

| **Tests** | **Week 0** (N=100) | **Week 24** (N=97) |
| --- | --- | --- |
| **Standard Progressive Matrices, n (%)** |  |  |
| Normal | 80 (80.0) | 81 (83.5) |
| Abnormal | 20 (20.0) | 16 (16.5) |
| Slow learner | 19 | 16 |
| Intellectual disability | 1 | - |
| **Coding** |  |  |
| Average to superior | 59 (59.0) | 66 (68.0) |
| Superior | 1 | 9 |
| Above average | 7 | 17 |
| Average | 51 | 40 |
| Below average | 41 (41.0) | 31 (32.0) |
| Below average | 25 | 24 |
| Far below average | 16 | 7 |
| **Trail Making Test: part A** |  |  |
| Average to fast | 46 (46.0) | 71 (73.2) |
| Fast | 21 | 42 |
| Average | 25 | 29 |
| Slow | 54 (54.0) | 26 (26.8) |
| **Trail Making Test: part B** |  |  |
| Average to fast | 31 (31.3) | 49 (51.0) |
| Fast | 13 | 32 |
| Average | 18 | 17 |
| Slow | 68 (68.7) | 47 (49.0) |
| **WCST^a^: total number of errors** | (N=98) | (N=96) |
| Average and above | 50 (51.0) | 59 (61.5) |
| Below average to impairment | 48 (49.0) | 1. 38.5) |

^a^WCST: Wisconsin Card Sorting Test

Executive function and global cognitive function by Wisconsin Card Sorting Test (WCST) evaluated at baseline and week 24 after switching efavirenz to rilpivirine in virologically-suppressed adolescents with HIV in Thailand

| Standard score | Week 0  (N=98) | Week 24  (N=96) |
| --- | --- | --- |
| Total number of errors | 92 (78-108) | 100 (83-115) |
| Perserverative responses | 96 (83-108) | 101 (85-122) |
| Perserverative errors | 99 (81-107) | 100 (85-122) |
| Percent conceptual level responses | 94 (78-104) | 102 (82-114) |
| Total number of errors: Level |  |  |
| Above average | 24 (24.5) | 33 (34.4) |
| Average | 26 (26.5) | 26 (27.1) |
| Below average | 21 (21.4) | 16 (16.7) |
| Mildly impaired | 10 (10.2) | 11 (11.5) |
| Mildly to moderately impaired | 5 (5.1) | 3 (3.1) |
| Moderately impaired | 7 (7.1) | 3 (3.1) |
| Moderately to severely impaired | 4 (4.1) | 1 (1.0) |
| Severely impaired | 1 (1.0) | 3 (3.1) |
| Perserverative responses: Level |  |  |
| Above average | 25 (25.5) | 37 (38.5) |
| Average | 32 (32.7) | 25 (26.0) |
| Below average | 17 (17.4) | 14 (14.6) |
| Mildly impaired | 11 (11.2) | 12 (12.5) |
| Mildly to moderately impaired | 5 (5.1) | 5 (5.2) |
| Moderately impaired | 1 (1.0) | 1 (1.0) |
| Moderately to severely impaired | 2 (2.0) | 1 (1.0) |
| Severely impaired | 5 (5.1) | 1 (1.0) |
| Perserverative errors: Level |  |  |
| Above average | 25 (25.5) | 41 (42.7) |
| Average | 34 (34.7) | 22 (22.9) |
| Below average | 13 (13.3) | 11 (11.5) |
| Mildly impaired | 11 (11.2) | 10 (10.4) |
| Mildly to moderately impaired | 7 (7.1) | 7 (7.3) |
| Moderately impaired | 1 (1.0) | 3 (3.1) |
| Moderately to severely impaired | 3 (3.1) | 2 (2.1) |
| Severely impaired | 4 (4.1) | - |
| Percent conceptual level responses: Level |  |  |
| Above average | 17 (17.4) | 34 (35.4) |
| Average | 35 (35.7) | 26 (27.1) |
| Below average | 17 (17.4) | 11 (11.5) |
| Mildly impaired | 12 (12.2) | 14 (14.6) |
| Mildly to moderately impaired | 5 (5.1) | 4 (4.2) |
| Moderately impaired | 9 (9.2) | 3 (3.1) |
| Moderately to severely impaired | 2 (2.0) | 1 (1.0) |
| Severely impaired | 1 (1.0) | 3 (3.1) |

Pediatric Quality of Life Inventory Version 4.0 (PedsQL): Parent report for children evaluated at baseline, week 4, and week 24 after switching efavirenz to rilpivirine in virologically-suppressed adolescents with HIV in Thailand (n=102)

| Parent report for children | Score; Median (IQR) | | |
| --- | --- | --- | --- |
|  | Week 0  (N=102) | Week 4  (N=101) | Week 24  (N=99) |
| Physical Functioning (problems with…) |  |  |  |
| 1. Walking more than one block | 0 (0-2) | 0 (0-2) | 0 (0-3) |
| 1. Running | 0 (0-2) | 0 (0-2) | 1 (0-2) |
| 1. Participating in sports activity or exercise | 1 (0-2) | 0 (0-2) | 1 (0-2) |
| 1. Lifting something heavy | 1 (0-2) | 1 (0-2) | 1 (0-2) |
| 1. Taking a bath or shower by him or herself | 0 (0-2) | 0 (0-3) | 0 (0-4) |
| 1. Doing chores around the house | 0 (0-2) | 0 (0-2) | 1 (0-2) |
| 1. Having hurts or aches | 0 (0-2) | 0 (0-1) | 1 (0-2) |
| 1. Low energy level | 0 (0-1) | 0 (0-1) | 0 (0-1) |
| Emotional Functioning (problems with…) |  |  |  |
| 1. Feeling afraid or scared | 0 (0-1) | 0 (0-1) | 0 (0-1) |
| 1. Feeling sad or blue | 0 (0-1) | 0 (0-1) | 0 (0-2) |
| 1. Felling angry | 1 (0-2) | 1 (0-2) | 1 (0-2) |
| 1. Trouble sleeping | 0 (0-2) | 0 (0-2) | 1 (0-2) |
| 1. Worrying about what will happen to him or her | 0 (0-2) | 0 (0-2) | 1 (0-2) |
| Social Functioning (problems with…) |  |  |  |
| 1. Getting along with other children | 0 (0-2) | 0 (0-2) | 0 (0-3) |
| 1. Other kids not wanting to be his or her friend | 0 (0-0) | 0 (0-0) | 0 (0-0) |
| 1. Getting teased by other children | 0 (0-1) | 0 (0-1) | 0 (0-0) |
| 1. Not able to do things that other children his or her age can do | 0 (0-0) | 0 (0-0) | 0 (0-0) |
| 1. Keeping up when playing with other children | 0 (0-1) | 0 (0-1) | 0 (0-1) |
| School Functioning (problems with…) |  |  |  |
| 1. Paying attention in class | 1 (0-2) | 1 (0-2) | 1 (0-2) |
| 1. Forgetting things | 2 (0-2) | 1 (0-2) | 2 (0-2) |
| 1. Keeping up with schoolwork | 1 (0-2) | 1 (0-2) | 1 (0-2) |
| 1. Missing school because of not feeling well | 0 (0-2) | 0 (0-1) | 1 (0-2) |
| 1. Missing school to go to the doctor or hospital | 1 (0-2) | 1 (0-2) | 2 (0-2) |

Pediatric Quality of Life Inventory Version 4.0 (PedsQL): Child report evaluated at baseline, week 4, and week 24 after switching efavirenz to rilpivirine in virologically-suppressed adolescents with HIV in Thailand (n=102)

| Child report | Score; Median (IQR) | | |
| --- | --- | --- | --- |
|  | Week 0  (N=102) | Week 4  (N=101) | Week 24  (N=99) |
| About My Health and Activities (problems with…) |  |  |  |
| 1. It is hard for me to walk more than one block | 0 (0-1) | 0 (0-1) | 0 (0-1) |
| 1. It is hard for me to run | 0 (0-1) | 0 (0-1) | 0 (0-1) |
| 1. It is hard for me to do sports activity or exercise | 0 (0-1) | 0 (0-1) | 0 (0-1) |
| 1. It is hard for me to lift something heavy | 1 (0-1) | 0 (0-1) | 0 (0-2) |
| 1. It is hard for me to take a bath or shower by myself | 0 (0-0) | 0 (0-0) | 0 (0-0) |
| 1. It is hard for me to do chores around the house | 0 (0-1) | 0 (0-1) | 0 (0-1) |
| 1. I hurt or ache | 0 (0-2) | 0 (0-1) | 0 (0-1) |
| 1. I have low energy | 0 (0-1) | 0 (0-1) | 0 (0-1) |
| About My Feelings (problems with…) |  |  |  |
| 1. I feel afraid or scared | 0 (0-1) | 0 (0-1) | 0 (0-1) |
| 1. I feel sad or blue | 0 (0-2) | 0 (0-1) | 1 (0-2) |
| 1. I feel angry | 1 (0-2) | 1 (0-2) | 1 (0-2) |
| 1. I have trouble sleeping | 1 (0-2) | 1 (0-2) | 1 (0-2) |
| 1. I worry about what will happen to me | 1 (0-2) | 0 (0-2) | 0 (0-2) |
| How I Get Along with Others (problems with…) |  |  |  |
| 1. I have trouble getting along with other kids | 0 (0-1) | 0 (0-1) | 0 (0-0) |
| 1. Other kids do not want to be my friend | 0 (0-0) | 0 (0-1) | 0 (0-0) |
| 1. Other kids tease me | 0 (0-1) | 0 (0-1) | 0 (0-1) |
| 1. I cannot do things that other kids my age can do | 0 (0-0) | 0 (0-0) | 0 (0-0) |
| 1. It is hard to keep up when I play with other kids | 0 (0-1) | 0 (0-1) | 0 (0-0) |
| About School (problems with…) |  |  |  |
| 1. It is hard to pay attention in class | 1 (0-2) | 1 (0-2) | 1 (0-2) |
| 1. I forget things | 2 (1-2) | 2 (1-2) | 1 (1-2) |
| 1. I have trouble keeping up with my schoolwork | 1 (0-2) | 1 (0-2) | 1 (0-2) |
| 1. I miss school because of not feeling well | 1 (0-2) | 1 (0-2) | 1 (0-1) |
| 1. I miss school to go to the doctor or hospital | 2 (0-2) | 2 (1-2) | 1 (0-2) |

Depression evaluated by Center for Epidemiologic Studies Depression Scale (CES-D) at baseline, week 4, and week 24 after switching efavirenz to rilpivirine in virologically-suppressed adolescents with HIV in Thailand (n=102)

| CES-D | Score; Median (IQR) | | |
| --- | --- | --- | --- |
|  | Week 0  (N=102) | Week 4  (N=101) | Week 24  (N=100) |
| 1. I was bothered by things that usually don’t bother me. | 1 (0-1) | 1 (0-1) | 1 (0-1) |
| 1. I did not feel like eating; my appetite was poor. | 0 (0-1) | 0 (0-1) | 1 (0-1) |
| 1. I felt that I could not shake off the blues even with help from my family or friends. | 0 (0-1) | 0 (0-1) | 0 (0-1) |
| 1. I felt I was just as good as other people. | 2 (1-2) | 2 (1-3) | 2 (1-3) |
| 1. I had trouble keeping my mind on what I was doing. | 0 (0-1) | 1 (0-1) | 1 (0-1) |
| 1. I felt depressed. | 0 (0-1) | 0 (0-1) | 0 (0-1) |
| 1. I felt that everything I did was an effort. | 0 (0-1) | 0 (0-1) | 0 (0-1) |
| 1. I felt hopeful about the future. | 2 (1-3) | 2 (1-3) | 2 (1-3) |
| 1. I thought my life had been a failure. | 0 (0-1) | 0 (0-1) | 0 (0-1) |
| 1. I felt fearful. | 0 (0-1) | 0 (0-1) | 0 (0-1) |
| 1. My sleep was restless. | 1 (0-1) | 0 (0-1) | 0 (0-1) |
| 1. I was happy. | 2 (2-3) | 2 (2-3) | 2 (2-3) |
| 1. I talked less than usual. | 0 (0-1) | 0 (0-1) | 0 (0-1) |
| 1. I felt lonely. | 0 (0-1) | 1 (0-1) | 0 (0-1) |
| 1. People were unfriendly. | 0 (0-0) | 0 (0-0) | 0 (0-0) |
| 1. I enjoyed life. | 2 (1-3) | 2 (2-3) | 2 (1-3) |
| 1. I had crying spells. | 0 (0-1) | 0 (0-1) | 0 (0-1) |
| 1. I felt sad. | 0 (0-1) | 0 (0-1) | 0 (0-1) |
| 1. I felt that people dislike me. | 0 (0-0) | 0 (0-1) | 0 (0-0) |
| 1. I could not get “going.” | 0 (0-1) | 0 (0-1) | 0 (0-1) |

Efavirenz-related symptoms assessed by a subject self-reported questionnaire evaluated at baseline, week 4, week 24, and week 48 after switching efavirenz to rilpivirine in virologically-suppressed adolescents with HIV in Thailand (n=102)

| Self-Report | Score; Median (IQR) | | | |
| --- | --- | --- | --- | --- |
|  | Week 0  (N=102) | Week 4  (N=101) | Week 24  (N=100) | Week 48  (N=97) |
| Nightmare | 0 (0-1) | 0 (0-1) | 0 (0-1) | 0 (0-1) |
| Trouble going to sleep | 0 (0-2) | 1 (0-2) | 1 (0-2) | 1 (0-1) |
| Waking up a lot | 0 (0-1) | 0 (0-1) | 0 (0-1) | 0 (0-1) |
| Restless sleep | 0 (0-1) | 0 (0-1) | 0 (0-1) | 0 (0-1) |
| Somnolence | 1 (0-2) | 1 (1-2) | 1 (1-2) | 1 (1-2) |
| Headache | 0 (0-1) | 1 (0-1) | 1 (0-1) | 1 (0-1) |
| Dizziness | 0 (0-1) | 0 (0-1) | 1 (0-1) | 0 (0-1) |
| Felt like the room was spinning | 0 (0-0) | 0 (0-0) | 0 (0-0) | 0 (0-0) |
| Unsteady walking | 0 (0-0) | 0 (0-0) | 0 (0-0) | 0 (0-0) |
| Impaired concentration | 1 (0-1) | 1 (0-1) | 1 (0-1) | 0 (0-1) |
| Amnesia | 1 (0-2) | 1 (1-2) | 1 (1-2) | 1 (1-2) |
| Agitation | 1 (0-2) | 1 (0-2) | 1 (0-2) | 1 (0-2) |
| Depression | 0 (0-1) | 0 (0-1) | 0 (0-1) | 0 (0-1) |
| Suicidality | 0 (0-0) | 0 (0-0) | 0 (0-0) | 0 (0-0) |
| Confusion | 0 (0-1) | 0 (0-1) | 0 (0-1) | 0 (0-1) |
| Hallucinations | 0 (0-0) | 0 (0-0) | 0 (0-0) | 0 (0-0) |
| Euphoria | 3 (2-3) | 3 (2-4) | 3 (2-4) | 2 (2-3) |
